# Trade-offs in seedling growth and survival within and across tropical forest microhabitats

**DOI:** 10.1002/ece3.1196

**Published:** 2014-09-09

**Authors:** Faith Inman-Narahari, Rebecca Ostertag, Gregory P Asner, Susan Cordell, Stephen P Hubbell, Lawren Sack

**Affiliations:** 1 Department of Ecology and Evolutionary Biology, University of California621 Charles E. Young Drive South, Los Angeles, California, 90095-1606; 2 Department of Biology, University of Hawaii200 W. Kawili Street, Hilo, Hawaii, 96720; 3 Department of Global Ecology, Carnegie Institution for Science260 Panama St., Stanford, California, 94305; 4 USDA Forest Service, Institute of Pacific Islands Forestry60 Nowelo Street, Hilo, Hawaii, 96720; 5 Center for Tropical Forest Science, Smithsonian Tropical Research InstituteBalboa, Republic of Panamá

**Keywords:** Determinants of plant community diversity and structure, light, performance rank changes, plant population and community dynamics, regeneration niche, relative growth rate, substrate, survival, topography, tropical forest diversity

## Abstract

For niche differences to maintain coexistence of sympatric species, each species must grow and/or survive better than each of the others in at least one set of conditions (i.e., performance trade-offs). However, the extent of niche differentiation in tropical forests remains highly debated. We present the first test of performance trade-offs for wild seedlings in a tropical forest. We measured seedling relative growth rate (RGR) and survival of four common native woody species across 18 light, substrate, and topography microhabitats over 2.5 years within Hawaiian montane wet forest, an ideal location due to its low species diversity and strong species habitat associations. All six species pairs exhibited significant performance trade-offs across microhabitats and for RGR versus survival within microhabitats. We also found some evidence of performance equivalence, with species pairs having similar performance in 26% of comparisons across microhabitats. Across species, survival under low light was generally positively associated with RGR under high light. When averaged over all species, topography (slope, aspect, and elevation) explained most of the variation in RGR attributable to microhabitat variables (51–53%) followed by substrate type (35–37%) and light (11–12%). However, the relative effects of microhabitat differed among species and RGR metric (i.e., RGR for height, biomass, or leaf area). These findings indicate that performance trade-offs among species during regeneration are common in low-diversity tropical forest, although other mechanisms may better explain the coexistence of species with small performance differences.

## Introduction

Ecologists debate the extent to which partitioning of regeneration niches, defined as differential performance of seeds and seedlings across environmental gradients, contributes to plant species coexistence (Grubb 1977; Chesson 2000; Wright 2002; Hubbell 2009). One criterion for niche differences to drive coexistence is that species must change ranks in performance (i.e., growth or survival) such that each species outperforms the others in at least one habitat condition (performance trade-offs), leading to differences in population growth rates among habitats (Abrams 1983; Chesson 2000; Wright 2002). Additionally, evidence has accumulated for a second type of trade-off, between growth and survival within and across habitats. For example, high relative growth rate (RGR) in high resource environments (e.g., high light) is associated with lower survival in low resource environments (e.g., low light; Kitajima 1994; Walters and Reich 1999; Sack and Grubb 2001; Wright et al. 2010). Some authors have argued that performance trade-offs across habitats are infrequent and make only a small contribution to tropical forest community assembly relative to growth–survival trade-offs because species' performance and habitat differences are small (Kitajima and Bolker 2003). An alternative hypothesis is that species within a community are functionally equivalent and that coexistence is maintained through random birth and death (i.e., neutral theory; Hubbell 2001). When species are ecologically equivalent (i.e., neither species outperforms the other in any situation; Hubbell 2001), “winners” at a particular site may be determined by neutral or other mechanisms such as density dependence (Janzen 1970; Connell 1971; Tilman 1994; Chesson 2000), dispersal limitation, or priority effects (i.e., who arrives first; Connell and Slatyer 1977; Urban and De Meester 2009). Although the concept of trade-offs is a cornerstone of niche theory, there remain very little data showing trade-offs for any habitat variables other than light.

Few field-based studies have clearly demonstrated that species differ in their regeneration niches such that seedlings change performance ranks (i.e., in RGR or survival) across habitats. Differential species responses to environmental variation and habitat associations have been well documented in tropical forests (e.g., Augspurger 1984; Wright et al. 2003; Engelbrecht et al. 2007), but most of these studies did not examine performance rank changes across habitats. Other trade-off studies have examined saplings or adult trees in the field (e.g., Pacala et al. 1994; Davies 2001), experimentally manipulated seedlings in greenhouses (e.g., Sack 2004), or employed experimental plantings rather than naturally established seedlings (Ashton and Gunatilleke 1995; Kobe 1999; Montgomery and Chazdon 2002; Baraloto et al. 2005; de Gouvenain et al. 2007). These data may not adequately represent adaptations of young plants in the wild (Bloor 2003; Cornelissen et al. 2003). Our approach complements and builds on previous studies of microhabitat trade-offs conducted with seeded or transplanted seedlings, noting that there are potential benefits and risks with either approach. Direct seeding and transplanting are reasonable approaches to looking at tradeoffs, especially once the “natural” habitat conditions have been defined through examination of wild populations. These methods have the advantage of controlling and balancing sample sizes. However, direct seeding and planting experiments risk introducing bias by, for instance, seeding or planting into sharply contrasting environments, such as very low versus very high light. This may exaggerate observed differences relative to the true differences shown by wild plants that are typically dispersed across a more continuous range of environmental conditions. Studies of wild seedlings may better represent the natural dynamics of forest regeneration, but results in unbalanced sample sizes and may not present clear contrasts among habitat conditions. We present our approach as a method that could potentially be employed by others given the existence of abundant data from other forests that would allow similar analyses (e.g., Comita and Engelbrecht 2009; Metz 2012).

We investigated performance trade-offs within and across microhabitats for seedlings of four common woody species in native-dominated Hawaiian montane wet forest. To our knowledge, this is the first study to examine performance trade-offs across multiple habitat dimensions for naturally established seedlings or for species in low-diversity tropical forest. We expected to find strong evidence of performance trade-offs in seedlings of common species in Hawaiian forest for four reasons. First, niche differences are thought to be most apparent during early tree regeneration stages because seedlings are more sensitive to environmental variation than adult trees (Grubb 1977; Poorter 2007). Second, assuming they are well-mixed spatially, common species interact with each other more frequently than do rare species and are therefore hypothesized to be more likely to evolve niche differences in response to interspecific competition (Hubbell 2006). Third, niche differences may be the primary coexistence mechanism in low-diversity forests because species interact with a smaller number of competitors which allows for niche displacement (as for common species) and because other coexistence mechanisms, such as negative density dependence and dispersal limitation, are predicted to be weaker (Janzen 1971; Hurtt and Pacala 1995; Gravel et al. 2006), although few studies have investigated these assumptions (Hille Ris Lambers et al. 2002; Johnson et al. 2012). Fourth, we measured multiple axes of microhabitat variation (light, substrate, and topography) and multiple growth variables (RGR for height, biomass, and leaf area) to achieve a high power to resolve species differences (Russo et al. 2012). Because of their extreme isolation and small land area, Hawaiian forests harbor fewer species than most tropical forests of comparable climate and structure (Carlquist 1985), thereby providing a unique model system for testing hypotheses about niche differentiation and coexistence in a low-diversity tropical forest. In a previous study, we showed that seedling distributions of common species are strongly limited by establishment and show strong habitat associations, suggesting that performance differences would be likely (Inman-Narahari et al. 2013).

We focused on seedling responses to three environmental factors – light, topography, and substrate – because these factors strongly affect plant growth and survival. First, light is often the most limiting resource for plant growth in tropical wet forests, and many studies demonstrate that plant species respond differentially to light gradients (e.g., Augspurger 1984; Kobe 1999; Sack and Grubb 2001; Montgomery and Chazdon 2002; Poorter and Arets 2003). Second, many plant species are strongly associated with topographic gradients (Comita and Engelbrecht 2009; Metz 2012). Topography typically correlates with essential plant growth resources such as soil moisture and nutrient availability with, for example, higher soil moisture availability on slopes than plateaus (Becker et al. 1988; Scowcroft and Jeffrey 1999; Daws et al. 2002; Sørensen et al. 2006). Third, we expected substrate to be an important axis of niche differentiation because many tree species show strong habitat associations with organic substrates such as logs and downed tree ferns; this is especially common in Hawaiian wet forest where feral ungulate soil disturbance is widespread (Cole et al. 2012; Murphy et al. 2014).

This study addresses the following questions: (1) Do species change ranks in their performance (RGR and survival) across habitats such that each species outperforms each of the others under some set of conditions (microhabitat trade-offs)? (2) Do species' RGR and survival ranks change within and across microhabitats (growth–survival trade-offs)? and (3) What is the relative importance of topography (aspect, slope, and elevation), light, and substrate microhabitats for seedling RGR?

## Materials and Methods

### Study site

We conducted this study in the 4 ha Laupahoehoe Forest Dynamics Plot (FDP), part of the Hawaii Permanent Plot Network (www.hippnet.hawaii.edu) and a member of the Smithsonian Tropical Research Institute Center for Tropical Forest Science (CTFS) network. The Laupahoehoe FDP is located on Hawaii Island (19°55′N, 155°17′W) in the Laupahoehoe Hawaii Experimental Tropical Forest (HETF). The FDP was established in 2008 at 1120 m elevation in native-dominated primary tropical lower montane wet forest (Holdridge 1947). The permanent plot is located in one of the few places in Hawaii where native forest dynamics can be studied because the presence of non-natives in this part of the forest is remarkably low and they are actively controlled. The Laupahoehoe FDP comprises 21 native woody species, including three tree fern species. The climate is largely aseasonal with 3440 mm mean annual rainfall (Giambelluca et al. 2013) and 16°C mean annual temperature (Juvik and Juvik 1998). Within the FDP, all native woody species ≥1 cm diameter at breast height (DBH, i.e., at 1.3 m) were tagged, mapped, measured, and identified following standard protocols applied throughout the CTFS plot network (Condit 1998).

### Data collection

In October–December 2008, we established a grid of 192 1 m × 1 m subplots within the 160 m × 160 m central area of the Laupahoehoe FDP following CTFS seedling plot protocols (Wright et al. 2005; Fig. S1). Within subplots, we tagged all native woody species <1 cm DBH, measured stem height from stem base to apex, and counted the number of leaves, including cotyledons (which are epigeal for these species). Measurements of small seedlings in the forest are subject to substantial error; thus, we rounded all measurements to the nearest 0.5 cm to equalize over- and underestimates. Following our initial census in November–December 2008, we remeasured previously tagged seedlings and measured new seedlings four times over 2.5 years: in December 2009, July 2010, December 2010–January 2011, and July 2011.

We examined the four species that were sufficiently abundant (*N *=* *30–1461) within seedling plots to analyze performance differences: *Metrosideros polymorpha*,*Cheirodendron trigynum*,*Coprosma rhynchocarpa*, and *Vaccinium calycinum* (nomenclature follows Wagner et al. 1999; we hereafter refer to these species by genus). These species vary substantially in growth form: *Metrosideros* is the dominant canopy tree in the Hawaiian Islands, *Cheirodendron* and *Coprosma* are midstory trees, and *Vaccinium* is an understory shrub. These four species comprised 98% of the seedlings found in seedling plots over the course of the study. They also represent 22% of the 18 species and 58% of the relative abundance (RA) of native woody species that reach ≥1 cm DBH in the Laupahoehoe FDP (Table1; RA and number of individuals for each species on each microhabitat category in Table S3).

**Table 1:** Information for the focal species analyzed in Hawaiian wet forest. Means ± SE with sample sizes in parentheses are shown. Species sharing the same letter are not significantly different based on one-way ANOVA with Tukey's HSD.”Wins” refers to the proportion of microhabitats in which each species performed significantly better than at least one other species.

Variable	*Cheirodendron trigynum*	*Coprosma rhynchocarpa*	*Metrosideros polymorpha*	*Vaccinium calycinum*
Family	Araliaceae	Rubiaceae	Myrtaceae	Ericaceae
Author	(Gaudich.) A. Heller	A.Gray	(H. Lév.) H. St. John	Sm.
Species code	*CT*	*CR*	*MP*	*VC*
Habit	Midstory tree	Midstory tree	Canopy tree	Understory shrub
Tree RA (%)	27	7.9	21	2.1
Tree RD (%)	6.2	0.87	38	0.049
Tree RF (%)	10.2	10.1	10.2	5.23
Mean initial height of seedlings (cm)	2.9 ± 0.04	3.81 ± 0.11	0.87 ± 0.04	4.04 ± 0.54
*N* for RGR/survival^1^	633/1461	153/342	807/1402	30/49
Seedling LMA (g·m^−2^)^2^	37^A^ ± 2.9 (11)	30^B^ ± 2.2 (14)	45^C^ ± 4.6 (19)	43^D^ ± 4.7 (7)
RGR_ht_ wins	0.04	0.27	0.69	0.67
RGR_pm_ wins	0.22	0.27	0.80	0.00
RGR_la_ wins	0.00	0.37	0.55	0.92
Survival wins	0.02	0.35	0.41	0.47
Mean wins for all metrics	0.07	0.31	0.61	0.51

Relative abundance (RA = number of individuals of species_i_/number of individuals of all species × 100), relative dominance (RD = basal area of individuals of species_i_/basal area of all species × 100), and relative frequency (RF = number of 20 × 20 m quadrates in which species was recorded/total number of quadrates × 100) are for trees ≥1 cm DBH within the 4 ha Laupahoehoe FDP.

1 Sample sizes were higher for survival than RGR because seedlings must have survived at least one census interval to calculate RGR.

2 Leaf mass per area (LMA = leaf mass/leaf area) calculated for seedlings harvested outside of FDP boundaries (*N *=* *5–21 individuals per species).

To evaluate topography, we used a digital elevation model (DEM) of the ground surface created by a small-footprint, high-power airborne Light Detection and Ranging (LiDAR) system developed by the Carnegie Airborne Observatory (Asner et al. 2007; Wu et al. 2012). For each seedling plot, we extracted slope, aspect, and elevation data from the DEM using the Spatial Analyst Tools in ArcGIS 10.0 (ESRI, Redlands, CA). To quantify light, we measured diffuse photosynthetically active radiation (PAR,*μ*mol photons·m^−2^·sec^−1^) four times at each seedling plot (Fig. S2) and paired these measurements with above-canopy PAR measurements to calculate transmitted PAR (TPAR) for each seedling plot (Nicotra and Chazdon 1994; Montgomery and Chazdon 2002). To compare RGR and survival of the four species across a variety of microhabitat conditions, we distinguished three discrete microhabitat categories (low, medium, and high) for the following continuous environmental variables: slope, elevation, and TPAR. For aspect, we identified three major categories using estimates of the proportion of land covered by each aspect (NE:0°-91°, SE-SW: 92°-242°, and W-NW:242°-360°). We used categories for our analysis rather than continuous variables because we did not expect to find linear responses to these variables. We recorded the rooting substrate of each seedling in six categories: live tree fern, dead tree fern, log, rock, root mat, and soil. Logs, tree ferns, and root mats are important rooting substrates for trees in Hawaiian forest (Santiago 2000). Detailed methods for topography and TPAR data collection are in Appendix S1. Category values, means, and variation in environmental conditions within the 4 ha FDP are in Appendix Table S1.

### Analysis

For each seedling, we calculated first year survival probability and three RGR metrics. Each metric provides insight into a different aspect of plant performance (Poorter et al. 2012). The first year survival probability is the survival of each seedling (survived = 1, died = 0) for the first year following the first measurement for that seedling. Thus, the probability of survival was counted only once for each seedling, only for the first year after it was first tagged. The three RGR metrics were as follows: (1) stem height RGR (RGR_ht_, cm·cm^−1^·year^−1^); (2) whole plant dry mass RGR (RGR_pm_, g·g·^−1 ^year^−1^); and (3) total leaf area RGR (RGR_la_, cm^2^·cm^−2^·year^−1^). We calculated RGR using the classic formula: RGR = (ln[final size] – ln[initial size])/(final date-initial date) (Hoffmann and Poorter 2002). Because relative and absolute growth rates typically change with size (Poorter and Garnier 1996; Paine et al. 2011), we equalized starting sizes by including only individuals with initial heights below 10 cm (mean initial heights for each species are listed in Table1).

We used species-specific allometric equations to estimate total plant biomass (above- and below-ground) and total plant leaf area from height and leaf counts for seedlings in seedling plots (after Montgomery and Chazdon 2002). We used whole plants harvested in the nearby forest outside the FDP to develop allometric equations to predict plant dry mass from height and to predict total leaf area from both the number of leaves and height. The*R*^2^ values were strong for regressions of mass versus height (0.80–0.96) and for leaf area versus height and number of leaves (0.91–0.99; Table S2). Detailed methods can be found in Appendix S1, regression equations are listed in Table S2, and plots of predicted versus actual values are shown in Figure S4A and B.

To test for RGR and survival differences among species within each microhabitat category, we used generalized linear mixed model analysis (GLMM) using the*lme4* R package (Bates and Maechler 2009). For all models, we included the subplot number as a random effect to account for spatial autocorrelation (Comita et al. 2010). Where the response variable was RGR, we used a Gaussian distribution, and where it was survival, we used a binomial distribution (i.e., logistic regression). To examine differences among species within a given microhabitat, we used Tukey's tests with*P*-values corrected for multiple comparisons using the Shaffer method in the*multcomp* R package (Shaffer 1986; Hothorn et al. 2008). To calculate mean RGR and survival for each species in each microhabitat category, we used the*lsmeans* R package (Lenth 2013) to calculate least squares means (LS-means) from the results of GLMM analysis. LS-means are recommended to provide a unbiased estimate for unbalanced designs (SAS OnlineDoc® 1999). For comparisons of performance in each microhabitat, we averaged values across all the other habitat categories (e.g., values for RGR for seedlings growing on fallen logs were compared averaging values for seedlings across all light and topography categories). Sample sizes would not permit analysis of species on combinations of variables. For GLMM analyses, we included species only where they were present in at least three subplots in each microhabitat category. We converted survival LS-means from log-odds ratios to probabilities for tables and figures.

We used the results of GLMM analysis to test for (1) microhabitat trade-offs for each species pair across microhabitats; (2) growth–survival trade-offs for each species pair within a given microhabitat; and (3) trade-offs between low-light survival and high-light RGR for each species pair (using low and high TPAR values). For a given species pair (e.g., species A vs. species B), where species A had higher performance (RGR or survival) in a given microhabitat than species B, we counted a “win” for species A. If species A performed better in some microhabitats (i.e., “wins”) but species B “wins” other microhabitats, we counted that as a microhabitat trade-off. Where we found no significant differences between species in a pair, we counted a “tie”. We counted a growth–survival trade-off where species A “wins” for RGR but species B “wins” for survival in a given microhabitat (or vice versa). Finally, we scored a growth–survival trade-off in high and low light if species pairs changed rank such that species A had higher growth in high-light but lower survival in low light than species B. Although species pairs were not independent, we use the number of species pairs with trade-offs to compare our results with other studies because this is a measure that is, available from most other studies on this topic.

To determine the effects of environmental factors on RGR, we conducted relative importance analysis for the contribution of topography, TPAR, and substrate. For this analysis, we included initial height as a covariate (using*relaimpo* R package; Lindeman et al. 1980; Gr mping 2006). We conducted all statistical analyses using R 3.0.1 (R Core Team 2013).

## Results

### Differences among species across all microhabitats

**Figure 1:**
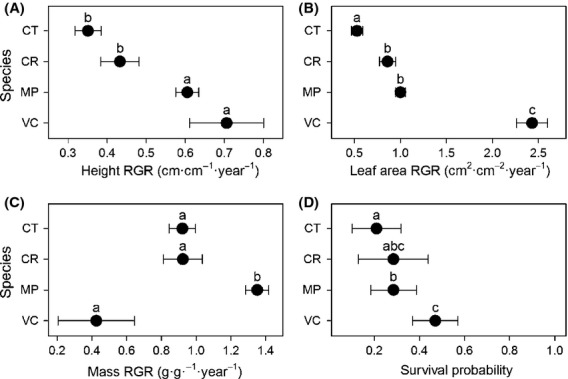
Mean relative growth rate (RGR) and survival probabilities for the four focal species averaged over all microhabitats in Hawaiian wet forest, (A) height RGR; (B) leaf area RGR; (C) dry mass RGR; and (D) survival; error bars represent SE; species sharing the same letter are not significantly different (GLMM analysis); species codes and sample sizes are in Table1. Note: although the error bars overlap for VC in panel D, the results of the mixed model analysis indicate significant differences.

Relative growth rates and survival differed among species when averaged across all microhabitats (Fig.1). These differences were reflected in the overall proportion of microhabitats “won” by each species (Table1). For example,*Vaccinium* had the highest RGR_la_ and survival and “won” 92% and 47% of microhabitats in species pair comparisons for RGR_la_ and survival, respectively.*Vaccinium* also had the lowest overall RGR_pm_ and “won” no microhabitats in species pair comparisons. Likewise,*Metrosideros* had the highest RGR_pm_ and “won” 80% of microhabitats for this RGR metric. Further,*Cheirodendron* had the lowest performance for all but RGR_pm_ and also “won” fewer habitats than the other species. Thus, overall performance across all microhabitats was a reasonable indicator of performance within each microhabitat.

### Microhabitat trade-offs

**Figure 2:**
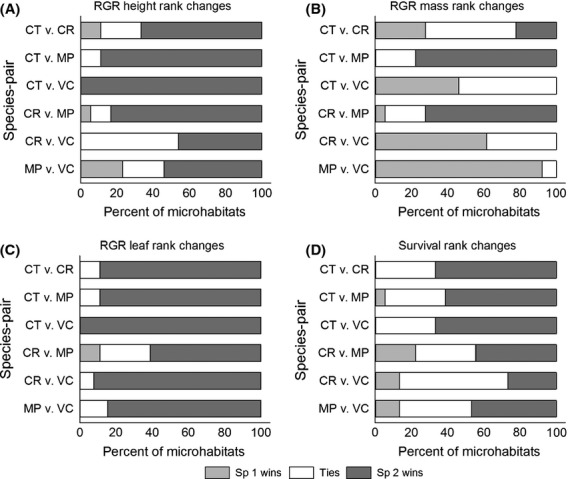
Species performance trade-offs across microhabitats for six species pairs of four common species in Hawaiian wet forest; bars represent the percent of microhabitats in which each species had significantly higher performance (A–C RGR and D survival) than the other species in the pair (listed in order of species 1 vs. species 2, e.g.,*MP* vs.*VC*) and in which neither species outperformed the other (i.e., ties); analysis includes from 13 to 18 light, substrate, and topography microhabitats with sufficient numbers of seedlings of each species; species codes and sample sizes are in Table1; detailed results in Appendix Table S4.

We found substantial evidence for shifts in species' relative performances across microhabitats for all six species pairs (Question #1; Fig.2, Table S3). Among the four focal species, even the worst performing species outperformed the best performing species in at least one microhabitat for at least one performance metric (Fig.2). Further, all species pairs showed equivalence in at least one microhabitat for at least one RGR metric (Fig.2). Indeed, the only species pair that showed no ties across microhabitats was*Cheirodendron* and*Vaccinium* for RGR_ht_ and RGR_la_.

### Growth–survival trade-offs

**Figure 3:**
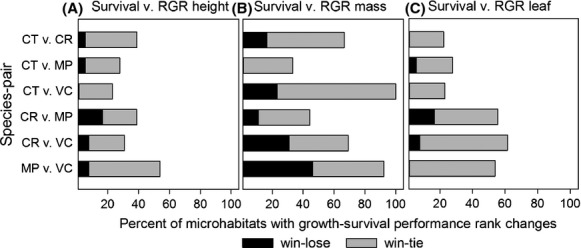
Growth–survival performance rank changes showing the proportion of microhabitats where RGR rank ≠ survival rank for each growth metric ((A)RGR_ht_, (B)RGR_pm_, and (C)RGR_la_); win–lose situations are those in which the species with the highest RGR did not also have higher survival or vice versa, win–tie situations are those in which one species in a pair had higher growth or survival, but did not differ from the other species in the other metric (e.g., higher growth and not different survival); analysis includes from 13 to 18 light, substrate, and topography microhabitats with sufficient numbers of seedling of each species; species codes and sample sizes are in Table1; detailed results in Appendix Table S4.

All species pairs showed growth–survival trade-offs within at least one microhabitat (Question #2; Fig.3). Three to five species pairs exhibited “win–lose” trade-offs. For example,*Cheirodendron* had higher RGR but lower survival than*Coprosma* on logs. However, all species pairs exhibited “win–tie” trade-offs. For example, species A had higher survival than species B, but the species did not differ in either RGR or survival within a particular habitat.

We found little evidence of trade-offs between survival in low TPAR and RGR in high TPAR. The general trend was for species with high RGR in high TPAR to also have high survival in low TPAR. However, for RGR_pm_, four of six species pairs changed ranks between RGR in high TPAR and survival in low TPAR (Fig. S5). For example,*Vaccinium* had the highest survival in low TPAR of the four species examined but had the lowest RGR_pm_ in high TPAR. For RGR_ht_, only one of six species pairs changed ranks between RGR in high TPAR and survival in low TPAR, such that*Metrosideros* had higher RGR_ht_ in high light than did*Vaccinium*, but lower survival in low TPAR (Fig. S5).

### Relative importance of microhabitat factors

**Figure 4:**
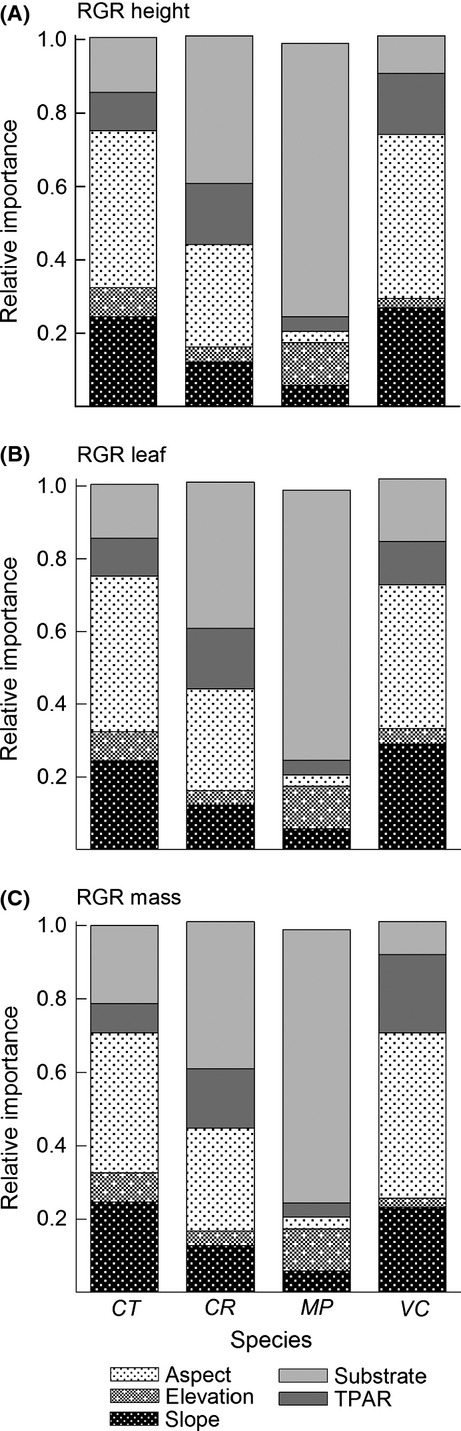
Relative importance of microhabitat characteristics for predicting (A) height, (B) leaf area, and (C) mass RGR for four common species in Hawaiian wet forest; calculated by partitioning*R*^2^ of generalized linear models after accounting for the effect of initial size; topography variables (aspect, elevation, and slope) are stippled; TPAR, transmitted photosynthetically active radiation.

The relative importance of microhabitat factors varied by RGR metric, although topography generally explained the largest proportion of variance in RGR that was attributable to the microhabitat variables we measured (Question #3; Fig.4). Averaged over all species, the microhabitat factors we measured explained 12–16% of the total variation in seedling RGR and initial height explained from 3% to 15%, depending on RGR metric. Of the variation partitioned among microhabitat variables, topography was the most important for all RGR metrics averaged over all species (51–53%). The second-most important factor was substrate (35–37%) followed by TPAR (11–12%).

The relative importance of habitat factors also varied among species. For example, topography explained 70–75% of microhabitat variation in RGR for*Cheirodendron*and*Vaccinium*, 43–44% of variation for *Coprosma*, and only 22% for *Metrosideros*. Additionally, for *Vaccinium*, the second-most important microhabitat variable differed among RGR metrics, with TPAR being more important than substrate for RGR_ht_ (17% vs. 10%) and RGR_la_ (21% vs. 9%), but with substrate being more important than TPAR for RGR_pm_ (12% vs. 17%). The relative importance of topographic attributes (slope, elevation, or aspect) also varied among species. Aspect was most important for *Cheirodendron*,*Coprosma*, and *Vaccinium* (28–45%), whereas elevation was most important for *Metrosideros* (11–12%; Fig.4).

## Discussion

### Strong performance differences across microhabitats

There was a surprising amount of performance differentiation among species, despite the broad one-dimensional habitat categories, suggesting that niches are important in this forest. Five of six species pairs exchanged ranks for mean RGR and/or survival in at least one microhabitat, supporting predictions of shifting performance hierarchies across habitats in low-diversity Hawaiian forest. In addition, two species “won” relatively few microhabitat categories (*Coprosma* and *Cheirodendron*) versus the other two species (*Metrosideros* and *Vaccinium*), indicating strong performance inequality between some species pairs. However, the relative proportion of “wins” for each species was not reflective of their relative abundance within the forest. Thus, it remains unclear whether these trade-offs drive coexistence.

Another key finding was the importance of what may be functional equivalence, represented as ties where neither species in a pair exhibited significant differences in RGR or survival. Because there were few habitats in which the poorest-performing species might “win” versus better-performing species, neutral mechanisms, such as priority effects and seed limitation (Chesson 2000; Hubbell 2001), may be more important for coexistence of the poorly performing species. For example, *Coprosma* was equivalent in performance to *Vaccinium* in 40% of microhabitats. These results suggest that microhabitat trade-offs at the regeneration stage occur in low-diversity Hawaiian wet forest, but the coexistence mechanism of some species pairs may be more likely due to random processes given that they showed little differentiation, whereas other species pairs may coexist via niche differences as they showed strong performance differences across microhabitats. Further work is needed to determine the extent to which performance differences contribute to species coexistence. We found a larger proportion of species pairs that differed in performance across habitats in Hawaii than has been reported for other forests by studies that used similar methods (Baraloto et al. 2005; Dent and Burslem 2009). For example, a study in French Guiana found performance differences across microhabitats for only three of 36 species pairs of seedlings transplanted across light and soil treatments (Baraloto et al. 2005). Another study in Borneo found no reversals for three species in a shade-house experiment comparing seedling growth across light and soil treatments (Dent and Burslem 2009). Other studies employing different analyses have also shown species' performance shifts across light levels, but did not quantify shifts by species pair (Agyeman et al. 1999; Sack and Grubb 2001). One explanation for the large proportion of trade-offs in Hawaiian forest is that species in low-diversity forests may be more likely to evolve niche differences because interspecific interactions are more predictable than in high-diversity forests (Hubbell 2006). Previous trade-off studies were conducted in high-diversity forests where hundreds of tree species coexist, whereas the forest where we conducted this study has only 21 woody species. Due to the low species diversity in Hawaiian forest, the four species we studied here represent a larger proportion of species diversity than in previous studies of performance trade-offs that were conducted in forests with higher species diversity. For example, of the hundreds of tree species in lowland wet forest in Costa Rica and French Guiana, Kobe (1999) and Baraloto et al. (2005) focused on four and nine species, respectively. Further, the species in this study all have low to no seed limitation (Inman-Narahari et al. 2013); low seed limitation is expected to increase the importance of niche relative to neutral mechanisms by increasing the potential for interspecific interactions (Hubbell 2001; Gravel et al. 2006). Another explanation is that we examined seedling responses to a larger number of habitat categories than most previous studies, which may increase the potential for discovering trade-offs (Kitajima and Poorter 2008; Philipson et al. 2011). In particular, the high-resolution topographic data provided by airborne LiDAR add a unique level of detail for examining species responses to topographic variation. These results point to the necessity of measuring several habitat characteristics across a range of forest types to further understand the extent of niche differentiation in tropical forests.

### Growth–survival trade-offs within microhabitats

We found substantial evidence of growth–survival trade-offs within given microhabitats. In the aforementioned study, only two of 36 species pairs showed “win–lose” growth–survival trade-offs within the same microhabitat (Baraloto et al. 2005). In our study, all species pairs exhibited “win–lose” growth–survival trade-offs. Comparing only for height growth, which was the only measurement reported by Baraloto et al. (2005), five of six species pairs showed “win–lose” growth–survival trade-offs. These growth–survival trade-offs often permitted the slower-growing species, (usually *Cheirodendron* or *Coprosma*), to “win” versus the faster-growing species, (usually *Metrosideros* or *Vaccinium*) by having higher survival. It appears that growth–survival trade-offs within microhabitats may be an important mechanism promoting coexistence and should be more widely investigated.

### Growth–survival trade-offs across light microhabitats

When examined across microhabitats, we found some evidence of trade-offs between RGR in high light versus survival in low light. The proportion of species pairs with trade-offs in Hawaiian forest was high relative to other forests for RGR_pm_, similar to other forests for RGR_ht_, and lower relative to other forests for RGR_la_. For example, Augspurger (1984) in Panama, Kobe (1999) in Costa Rica, and Baraloto et al. (2005) in French Guiana found high-light RGR versus low-light survival trade-offs for 14%, 33%, and 22% of species pairs, respectively. In our study, four of six species pairs showed a negative correlation between high-light RGR_pm_ versus low-light survival (60%), but only one pair showed such trade-offs for RGR_ht_ and none for RGR_la_. The results for RGR_pm_ are consistent with the theory of a general trade-off in physiological capabilities between shade tolerators and light demanders (Kitajima 1994; Sack and Grubb 2001; Kitajima and Poorter 2008), but results for the other growth metrics do not support this theory.

### Relative importance of microhabitat factors

The relative importance analysis showed that substrate type and topographic variables were important determinants of growth rates for these species. Topography, especially slope and aspect, was the most important environmental variable for predicting seedling RGR for most species. Slope and aspect strongly affect forest soil resources (e.g., moisture and nutrients), temperature, and litter thickness (Daws et al. 2002; John et al. 2007), which in turn strongly and differentially influence species growth (Palmiotto et al. 2004; Engelbrecht et al. 2007; Metz 2012). The relative importance of topography suggests that soil resources and other factors associated with topography are stronger drivers of seedling RGR than either light or substrate for most species in this forest. Light was less important in this forest, but we include it for comparative purposes as it is an important factor in many other forests and is one of the environmental variables most frequently investigated. The relatively weak role of light in explaining seedling RGR is consistent with the relatively higher understory light in Hawaiian forests (Inman-Narahari et al. 2013) and with a recent study reporting that light availability explained <12% of variation in tree growth rates within a lowland tropical forest in Panama (Rüger et al. 2011). For the dominant canopy species, *Metrosideros*, substrate type was the most important microhabitat variable. Disconcertingly, the substrate type on which *Metrosideros* had the lowest RGR and survival was dead tree ferns, on which they are frequently found growing (Inman-Narahari et al. 2013). The relative importance of microhabitat factors requires more investigation to understand how plant available resources vary with topography and substrate and the physiological mechanisms by which these resources affect seedling RGR in forests.

### Performance metrics matter

An important finding of this study was that interpretations of habitat trade-offs can depend on which performance metrics are measured. We included the three growth metrics to provide a better understanding of the ways in which plant growth differs for these species under the various habitat conditions we examined. Height growth (the most commonly measured variable for most seedling studies) provides information about growth only in one dimension, whereas plants actually grow in multiple dimensions. For example, leaf area growth may be more reflective of plant adaptations to low light in the forest understory, and biomass growth is indicative of mass balance of the whole plant, including the roots. Although we accept that our modeled approach is imperfect, it still provides intriguing clues that these variables should be considered, rather than strict reliance on height growth measurements, as is commonly performed. For example, if we had restricted our analysis to only leaf area RGR, we would have found microhabitat performance tradeoffs for only one of the six species pairs. Instead, we found 3/6, 2/6, 1/6, and 4/6 species pairs that had tradeoffs for RGR height, RGR mass, RGR leaf area, and survival, respectively. Similarly, a recent study in tropical China found trade-offs for mass but not height growth metrics (Yang et al. 2011), and an experiment on seedling responses to regional environmental gradients in Panama found stronger responses for leaf area than height (Brenes-Arguedas et al. 2011). We found the most microhabitat performance tradeoffs for survival. To our knowledge, this is the first time that three different RGR metrics and survival have been used to analyze species performance differences across microhabitats. The fact that all these variables are examined less frequently in studies of performance differences across habitats might partially explain the large proportion of trade-offs found in this study compared with previous research (Kobe 1999; Baraloto et al. 2005; Dent and Burslem 2009).

Different performance metrics may provide different insights into plant population dynamics. For example, rapid height growth may lead to increased light interception and eventually contribute to competitive dominance where vertical light gradients are very steep (Givnish 1982). However, leaf area growth can likewise increase light interception and correspond to photosynthetic area for potential carbon gain (Koyama and Kikuzawa 2009). This may be more important for small seedlings because their growth is more restricted by their ability to acquire limited resources in the understory than by direct competition with one another (Paine et al. 2008). Although the relationships between plant growth and population growth rates are unclear, we expect that, all else being equal, high survival would be closely correlated with high population growth rates. Given the importance of growth–survival trade-offs, survival appears to be an especially important performance metric to elucidate niche differentiation. As each metric may potentially vary in importance for different aspects of a species' performance, the most comprehensive approach is to measure several aspects of plant performance to determine interspecific differences.

### Caveats regarding coexistence

Our findings indicate that differential responses to microhabitats during regeneration in low-diversity tropical forest fulfill the theoretical requirements of niche differentiation, a mechanism that may contribute to species coexistence. However, making inferences about community assembly and coexistence based on seedling performance data remains challenging, especially given that seedlings provide information on only a short span of a trees' lifetime. Nevertheless, some trade-off patterns were consistent with the observed species relative abundance in the Laupahoehoe FDP. For example, *Metrosideros* had higher RGR than the three other species examined in the majority of conditions, consistent with it being the dominant canopy tree in this Hawaiian montane wet forest (Asner et al. 2009; Vitousek et al. 2009). On the other hand, *Cheirodendron*, a midstory tree species with lower RGR, had higher relative abundance within the 4 ha plot than did *Metrosideros* (Table1), suggesting that adult size differences may be an aspect of significant niche differentiation which occur at later life stages that our analysis did not take into account (Baraloto et al. 2005). Though impractical for this study due to our sample sizes, further examination of seedling trade-offs could consider seedling RGR under combinations of given factors (e.g., on logs in high TPAR versus logs in low TPAR). Nevertheless, our data support the concept that niche differentiation, in concert with other mechanisms, is a potential contributor to patterns of forest dominance for endemic tree species in Hawaiian montane wet forest. Future work is needed to clarify whether niche mechanisms primarily determine coexistence among the strongest competitors with neutral or other mechanisms being more important for coexistence among the least competitive species.

## Supporting Information

**Appendix S1.**Additional details on data collection and analysis methods. **Table S1.** Microhabitat factors across the 4-ha Forest Dynamics Plot (FDP) in Hawaiian wet forest with mean ± SE, range and percent cover within categories. **Table S2.** Allometric equations and results of linear regression analysis used for estimating plant dry mass and leaf area for seedlings in forest plots in Hawaiian wet forest; mean values and ranges of harvested seedlings used for regressions; for each species, we harvested 8–20 individuals across a range of sizes (0.85–63 cm) and light habitats (1.7–15%; mean: 5.0%) corresponding to those found in seedling plots. **Table S3.** Number of seedlings and relative abundance (RA) for each species in each microhabitat category. **Table S4.** (A–D) Differences among species in relative growth rates (RGR) and survival probability on each habitat category calculated with least squares means (Means) and standard errors (SE) from GLMM analysis. **Figure S1.** (A) Schematic of census station locations within the 4-ha forest dynamics plot in Hawaiian native forest, and (B) diagram of a census station with three 1-m^2^ seedling subplots within 2 m of a 0.5-m² seed trap. **Figure S2.** Mean TPAR at each census interval in Hawaiian wet forest, letters based on Tukey's HSD analysis of repeated measures ANOVA; error bars represent SE, TPAR mean (6.4%) and SE over all four censuses represented by dashed horizontal line and represented by dotted horizontal lines, respectively. **Figure S3.** For each species, left plot shows natural log transformed height data over time (days since first measurement) and right plot shows natural log transformed mean heights versus mean days at each census (*N *=* *5 censuses). Solid lines represent log-linear models and dashed lines represent lowess smoothed lines. Model fits show that log-linear models provide a reasonable approximation of growth for seedling species over the time period examined here. **Figure S4.** (A, B) Scatter plots of actual versus predicted plant mass of seedlings used for allometric equations (see Table S2 for full equations). **Figure S5.** Trade-offs between RGR in high TPAR and survival in low TPAR showing species rank changes in each category (species abbreviations as in Table1).
