# Supplementary material for: Trade-offs in seedling growth and survival within and across tropical forest microhabitats
Source: Ecol Evol. 2014 Sep 9;4(19):3755–67. doi: 10.1002/ece3.1196 (PMC4301042; doi:10.1002/ece3.1196)
Supplement: Supplementary file 1 — Appendix S1.Additional details on data collection and analysis methods. Table S1. Microhabitat factors across the 4-ha Forest Dynamics Plot (FDP) in Hawaiian wet forest with mean ± SE, range and percent cover within categories. Table S2. Allometric equations and results of linear regression analysis used for estimating plant dry mass and leaf area for seedlings in forest plots in Hawaiian wet forest; mean values and ranges of harvested seedlings used for regressions; for each species, we harvested 8–20 individuals across a range of sizes (0.85–63 cm) and light habitats (1.7–15%; mean: 5.0%) corresponding to those found in seedling plots. Table S3. Number of seedlings and relative abundance (RA) for each species in each microhabitat category. Table S4. (A–D) Differences among species in relative growth rates (RGR) and survival probability on each habitat category calculated with least squares means (Means) and standard errors (SE) from GLMM analysis. Figure S1. (A) Schematic of census station locations within the 4-ha forest dynamics plot in Hawaiian native forest, and (B) diagram of a census station with three 1-m2 seedling subplots within 2 m of a 0.5-m² seed trap. Figure S2. Mean TPAR at each census interval in Hawaiian wet forest, letters based on Tukey's HSD analysis of repeated measures ANOVA; error bars represent SE, TPAR mean (6.4%) and SE over all four censuses represented by dashed horizontal line and represented by dotted horizontal lines, respectively. Figure S3. For each species, left plot shows natural log transformed height data over time (days since first measurement) and right plot shows natural log transformed mean heights versus mean days at each census (N = 5 censuses). Solid lines represent log-linear models and dashed lines represent lowess smoothed lines. Model fits show that log-linear models provide a reasonable approximation of growth for seedling species over the time period examined here. Figure S4. (A, B) Scatter plots of actual versus pr [file ece30004-3755-sd1.pdf]

APPENDICES

Appendix S1: Additional details on data collection and analysis methods

Topography data collection

We used high resolution LiDAR data to measure microtopography in seedling plots. The onboard Carnegie Airborne Observatory Alpha System recorded 100,000-Hz LiDAR pulse repetition frequency with a laser beam divergence of 0.56-mrad flown at 1000-m above ground level, holding relative distance to ground constant across the plot (Asner et al. 2007). Multiple adjacent flight lines mapped the area with 50% overlap, thereby achieving at least two laser shots per 1.12-m × 1.12-m area. We ran the LiDAR with full waveform digitization, providing the detailed vertical canopy profile information necessary to detect plant cover down to an effective spatial resolution of 0.56-m × 0.56-m (Wu et al. 2012). We modeled the ground DEM using a non-linear interpolation routine that seeks lowest reflected return energy in each laser waveform, after normalizing the waveforms in each 1.12-m resolution pixel to nadir (Wu et al. 2012). When we received no ground-level data (*e.g.*, when a large tree stem stopped laser energy), we estimated a ground point by triangulated network modeling. We smoothed the resulting DEM with a 3 × 3 moving kernel.

Transmitted photosynthetically active radiation (TPAR) data collection

To quantify irradiance within seedling plots, we recorded two 15-sec averages of understory photosynthetically active radiation (PAR, μmol photons$\cdot$m$^{-2}\cdot$s^{-1}) on uniformly overcast days using a 1-m long line quantum sensor (LI-191, LI-COR, Lincoln, NE, USA) positioned 1-m

above the center of each seedling plot. We paired understory values with simultaneous above-canopy PAR measurements logged at 1-minute intervals by a point quantum sensor (LI-190; LI-COR) mounted on an above-canopy climate tower located within 200-m of the FDP. We calculated transmitted PAR (TPAR) as understory PAR / above canopy PAR (Anderson 1964, Nicotra and Chazdon 1994, Montgomery and Chazdon 2002). We measured TPAR four times, in December 2009, July 2010, December 2010, and June 2011 (Fig. S2), using the average across all measurement times in our subsequent analysis.

Categorization methods for continuous microhabitat variables

We categorized continuous microhabitat variables (slope, aspect, elevation and TPAR) into three categories using quantiles for each variable. This categorization method provided good representation of the variability within the larger 4-ha plot as assessed using GIS maps and also provided adequate sample sizes of seedling plots in each category. Resulting categories are listed in Appendix Table S1. The rationale for using categories rather than continuous variables was that we did not expect seedling performance to respond linearly to any of the variables.

Calculations of plant mass and total leaf area

We estimated total plant dry mass (above and belowground) and total leaf area from species-specific linear regressions of plant dry mass versus height or leaf area versus height, respectively, from whole plants harvested in the nearby forest outside the FDP (Montgomery and Chazdon 2002). Because understory irradiance can affect leaf traits, we harvested seedlings across a range of understory irradiance microhabitats; tests for a relationship between TPAR and leaf traits for these seedlings were non-significant at $\alpha = 0.05$.

Relative growth rate model tests

This RGR model yields a single RGR parameter integrating over the growing period, while true RGR declines ontogenetically and fluctuates diurnally and seasonally with environmental sensitivity. However, an integrated RGR is appropriate to compare seedlings in a given growth period, especially for long lived trees (Paine *et al.* 2011). To test this assumption, we examined fits of a logistic growth models for each species using seedlings with at least four census measurements (a small subset of the total number of seedlings measured). This analysis showed that log transformed height increased roughly linearly with time, consistent with RGR remaining stable with time over the measurement period (Fig. S3). Thus, we felt confident that the classic RGR model was acceptable for our data.

Table S1. Microhabitat factors across the 4-ha Forest Dynamics Plot (FDP) in Hawaiian wet forest with mean ±SE, range and percent cover within categories. We quantified percent cover of topography categories as the proportion of seedling subplots in each category, excluding subplots without seedlings (*N* = 153). We quantified the percentage cover of each substrate within the 4-ha plot using 20-m point-intercept transects sampled every 10 cm (*N* = 32)

	Mean ±SE	Range	Microhabitat Categories	Percent cover of categories
Slope (°)	15 ±0.066	0 - 60	Low: 0-8.24	22
			Medium: 8.24-18.14	39
			High: 18.15-60.00	39
Elevation (m)	1169 ±0.04	1159 - 1188	Low: 1159-1164	42
			Medium: 1165-1172	37
			High: 1172-1188	22
TPAR (%)	6.4 ±0.29	1.4 - 26.9	Low: 1.4-4.0	48
			Medium: 4.1-7.0	60
			High 7.1-26.9	45
Aspect (°)	166 ±0.74	0 - 360	NE (0-91)	34
			SE-SW (92-242)	31
			W-NW (242-360)	20
Substrate			Dead tree fern	6.5 ±1.0
			Live tree fern	2.3 ±0.44
			Log	3.9 ±0.84
			Rock	8.8 ±1.4
			Root mat	32 ±2.9
			Soil	46 ±3.3

Table S2. Allometric equations and results of linear regression analysis used for estimating plant dry mass and leaf area for seedlings in forest plots in Hawaiian wet forest; mean values and ranges of harvested seedlings used for regressions; for each species, we harvested 8-20 individuals across a range of sizes (0.85- 63 cm) and light habitats (1.7% - 15%; mean: 5.0%) corresponding to those found in seedling plots

		CT	*CR*	*MP*	*VC*
Height (cm)	N	7	14	19	7
	Mean	13.5	21.4	16.6	32.7
	Min.	4.7	1.2	0.82	12.5
	Max.	22	62.9	45.8	44.7
Dry mass (g)	Mean	0.54	1.91	1.12	1.86
	Min.	0.018	0.0026	0.00084	0.24
	Max.	1.66	7.18	5.95	4.15
Leaf area (cm^2)	Mean	60	202	80.20	101
	Min.	3.41	0.32	0.24	4.84
	Max.	158	608	367	203
Dry mass regression equation: log(dry mass)=	Intercept (a)	-8.10	-6.34	-6.87	-2.68
a+b×log(height)	Slope (b)	2.69	2.13	2.22	0.09
	R^2	0.93	0.94	0.96	0.80
Leaf area regression	Intercept	-1.79	-0.99	-1.02	-7.39
equation: log(leaf area)=	Slope (b_1)	1.65	2.01	1.65	3.71
$a+b_1\times\log(height)+b_2\times num.$	Slope (b_2)	0.10	-0.01	0.01	-0.01
leaves	R^2	0.99	0.91	0.95	0.92

Table S3. Number of seedlings and relative abundance (RA) for each species in each microhabitat category.

	N				RA			
Habitat Category	CT	CR	MP	VC	CT	CR	MP	VC
Dead tree fern	250	22	190	6	0.53	0.05	0.41	0.01
Log	61	6	55	1	0.5	0.05	0.45	0.01
Live tree fern	134	7	108	6	0.53	0.03	0.42	0.02
Root mat	169	39	349	13	0.3	0.07	0.61	0.02
Rock	10	16	56	3	0.12	0.19	0.66	0.04
Soil	9	62	48	1	0.08	0.52	0.4	0.01
Elev low	152	23	104	2	0.54	0.08	0.37	0.01
Elev med	219	91	432	13	0.29	0.12	0.57	0.02
Elev high	262	39	271	15	0.45	0.07	0.46	0.03
Slope low	36	45	79	1	0.22	0.28	0.49	0.01
Slope med	134	54	241	8	0.31	0.12	0.55	0.02
Slope high	463	54	487	21	0.45	0.05	0.48	0.02
Aspect low	253	39	219	5	0.49	0.08	0.42	0.01
Aspect med	188	59	289	15	0.34	0.11	0.52	0.03
Aspect high	192	55	299	10	0.35	0.1	0.54	0.02
TPAR low	91	47	111	6	0.36	0.18	0.44	0.02
TPAR med	301	66	344	16	0.41	0.09	0.47	0.02
TPAR high	241	40	352	8	0.38	0.06	0.55	0.01

Table S4 A-D Differences among species in relative growth rates (RGR) and survival probability on each habitat category calculated with least squares means (Means) and standard errors (SE) from GLMM analysis. Significant differences represented by different letters. Species codes in Table 1. Empty cells represent habitat categories with sample sizes too low to analyze.

S4 A. Height relative growth rate (RGR_{ht}, $cm \cdot cm^{-1} \cdot yr^{-1}$)

Habitat Category	CT	CR	MP	VC	Mean CT	SE CT	Mean CR	SE CR	Mean MP	SE MP	Mean VC	SE VC
Dead tree fern	a	ab	b	b	0.22	0.06	0.45	0.15	0.41	0.06	0.77	0.19
Log	a	ab	b		0.39	0.12	0.38	0.28	0.79	0.12		
Live tree fern	a	ab	b	ab	0.34	0.06	0.28	0.19	0.70	0.06	0.76	0.21
Root mat	a	ab	b	ab	0.35	0.06	0.41	0.08	0.50	0.04	0.62	0.13
Rock	a	a	a		0.37	0.23	0.40	0.19	0.82	0.12		
Soil	a	a	b		0.27	0.16	0.49	0.06	0.76	0.08		
Elev low	a	ab	b		0.44	0.10	0.48	0.14	0.77	0.10		
Elev med	a	ab	b	ab	0.35	0.05	0.46	0.07	0.61	0.04	0.59	0.15
Elev high	a	ab	b	b	0.32	0.05	0.38	0.08	0.54	0.04	0.72	0.13
Slope low	a	a	a		0.40	0.12	0.48	0.11	0.71	0.09		
Slope med	a	ab	b	ab	0.27	0.07	0.39	0.09	0.60	0.06	0.63	0.20
Slope high	a	ab	b	b	0.38	0.04	0.45	0.07	0.60	0.04	0.76	0.11
Aspect low	a	ab	b	ab	0.31	0.05	0.35	0.08	0.58	0.05	0.70	0.22
Aspect med	a	ab	bc	c	0.39	0.05	0.42	0.08	0.55	0.04	0.86	0.13
Aspect high	a	ab	b	ab	0.38	0.08	0.52	0.09	0.69	0.07	0.48	0.18
TPAR low	a	ab	b	ab	0.28	0.07	0.45	0.09	0.55	0.07	0.58	0.23
TPAR med	a	ab	bc	c	0.36	0.05	0.45	0.08	0.59	0.04	0.84	0.13
TPAR high	a	a	b	ab	0.38	0.06	0.41	0.09	0.66	0.05	0.51	0.18

S4 B. Plant mass relative growth rate (RGR_{pm})

Habitat Category	CT	CR	MP	VC	Mean CT	SE CT	Mean CR	SE CR	Mean MP	SE MP	Mean VC	SE VC
Dead tree fern	a	ab	b	ab	0.60	0.14	0.95	0.34	0.92	0.13	0.35	0.45
Log	a	a	a		1.03	0.28	0.82	0.65	1.75	0.29		
Live tree fern	a	ab	b	ab	0.91	0.14	0.58	0.45	1.55	0.13	0.40	0.49
Root mat	ab	ab	b	a	0.94	0.13	0.87	0.19	1.12	0.09	0.19	0.31
Rock	a	a	a		0.97	0.51	0.86	0.43	1.82	0.27		
Soil	a	a	b		0.69	0.35	1.04	0.14	1.70	0.18		
Elev low	a	ab	b		1.13	0.23	1.00	0.31	1.70	0.22		
Elev med	a	ab	b	a	0.92	0.12	0.98	0.16	1.35	0.10	0.36	0.34
Elev high	a	ab	b	a	0.84	0.10	0.80	0.19	1.21	0.10	0.36	0.31
Slope low	a	a	a		1.09	0.28	1.02	0.26	1.58	0.21		
Slope med	a	ab	b	ab	0.74	0.15	0.83	0.19	1.33	0.12	0.26	0.46
Slope high	a	ab	b	a	0.98	0.10	0.97	0.17	1.33	0.09	0.50	0.25
Aspect low	b	b	a	ab	0.85	0.12	0.74	0.20	1.30	0.12	0.42	0.52
Aspect med	ab	ab	b	a	1.02	0.11	0.90	0.18	1.24	0.09	0.45	0.30
Aspect high	a	ab	b	a	0.94	0.17	1.11	0.21	1.53	0.15	0.43	0.41
TPAR low	a	a	a	a	0.76	0.17	0.95	0.21	1.22	0.15	0.44	0.54
TPAR med	a	ab	b	a	0.96	0.11	0.96	0.17	1.31	0.10	0.47	0.30
TPAR high	a	a	b	a	0.98	0.14	0.86	0.21	1.46	0.12	0.31	0.42

S4 C. Leaf area relative growth rate (RGR_{la})

Habitat Category	CT	CR	MP	VC	Mean CT	SE CT	Means CR	SE CR	Mean MP	SE MP	Mean VC	SE VC
Dead tree fern	a	ab	b	c	0.26	0.10	0.90	0.26	0.68	0.10	2.59	0.33
Log	a	ab	b		0.64	0.20	0.74	0.46	1.31	0.21		
Live tree fern	a	ab	b	c	0.53	0.11	0.55	0.35	1.18	0.11	2.74	0.38
Root mat	a	ab	b	c	0.52	0.10	0.81	0.15	0.80	0.08	2.34	0.24
Rock	a	a	a		0.63	0.39	0.81	0.33	1.36	0.21		
Soil	a	ab	b		0.48	0.30	0.96	0.12	1.27	0.16		
Elev low	a	ab	b		0.69	0.19	0.94	0.24	1.29	0.18		
Elev med	a	b	b	c	0.53	0.09	0.90	0.12	1.00	0.08	2.00	0.26
Elev high	a	ab	b	c	0.48	0.09	0.77	0.15	0.90	0.08	2.59	0.24
Slope low	a	a	a		0.63	0.21	0.96	0.21	1.18	0.17		
Slope med	a	ab	b	c	0.40	0.12	0.77	0.15	0.99	0.10	2.22	0.36
Slope high	a	b	b	c	0.57	0.08	0.91	0.13	0.98	0.07	2.58	0.19
Aspect low	a	ab	b	c	0.46	0.10	0.70	0.15	0.97	0.09	2.30	0.38
Aspect med	a	ab	b	c	0.59	0.09	0.83	0.14	0.90	0.08	3.08	0.23
Aspect high	a	ab	b	b	0.59	0.14	1.02	0.16	1.16	0.12	1.52	0.31
TPAR low	a	b	b	b	0.40	0.13	0.88	0.16	0.94	0.12	1.79	0.40
TPAR med	a	ab	b	c	0.55	0.09	0.90	0.14	0.97	0.08	2.98	0.23
TPAR high	a	ab	bc	c	0.59	0.11	0.81	0.16	1.08	0.09	1.79	0.32

S4 D. Survival

Habitat Category	CT	CR	MP	VC	Mean CT	SE CT	Means CR	SE CR	Mean MP	SE MP	Mean VC	SE VC
Dead tree fern	a	b	ab	ab	-2.03	0.32	-0.32	0.65	-1.97	0.33	-1.00	0.75
Log	ab	a	b	ab	-1.27	0.37	0.14	0.67	-1.92	0.40	-15.89	975.90
Live tree fern	a	a	a	a	-0.99	0.40	-1.18	0.79	-0.37	0.39	0.93	1.05
Root mat	a	ab	b	ab	-1.44	0.24	-1.00	0.33	-0.70	0.20	-0.20	0.51
Rock	a	a	a		-1.77	0.53	-0.64	0.49	-1.00	0.34		
Soil	a	b	c		-2.67	0.64	-1.04	0.28	1.32	0.48		
Elev low	a	a	a		-1.27	0.29	-0.68	0.40	-1.39	0.30		
Elev med	a	ab	b	b	-1.41	0.15	-0.89	0.20	-0.85	0.13	-0.20	0.43
Elev high	a	ab	b	ab	-1.36	0.19	-1.14	0.29	-0.78	0.19	-0.16	0.50
Slope low	a	a	a	a	-1.38	0.33	-0.85	0.36	-0.88	0.29	-1.16	1.28
Slope med	a	a	a	a	-1.42	0.19	-1.16	0.25	-0.95	0.17	-0.92	0.65
Slope high	a	abc	b	c	-1.25	0.15	-0.76	0.24	-0.88	0.14	0.32	0.41
Aspect low	a	a	a	a	-1.32	0.21	-0.72	0.31	-1.13	0.20	-0.23	0.68
Aspect med	a	ab	b	c	-1.23	0.15	-0.79	0.24	-0.65	0.14	0.66	0.48
Aspect high	a	ab	b	ab	-1.53	0.22	-1.17	0.27	-1.01	0.19	-1.15	0.61
TPAR low	a	ab	b	ab	-1.69	0.23	-1.37	0.30	-1.00	0.23	-0.54	0.78
TPAR med	a	bc	ab	c	-1.14	0.17	-0.32	0.25	-0.88	0.15	0.56	0.49
TPAR3 high	a	ab	b	ab	-1.31	0.18	-1.20	0.27	-0.87	0.16	-0.71	0.54

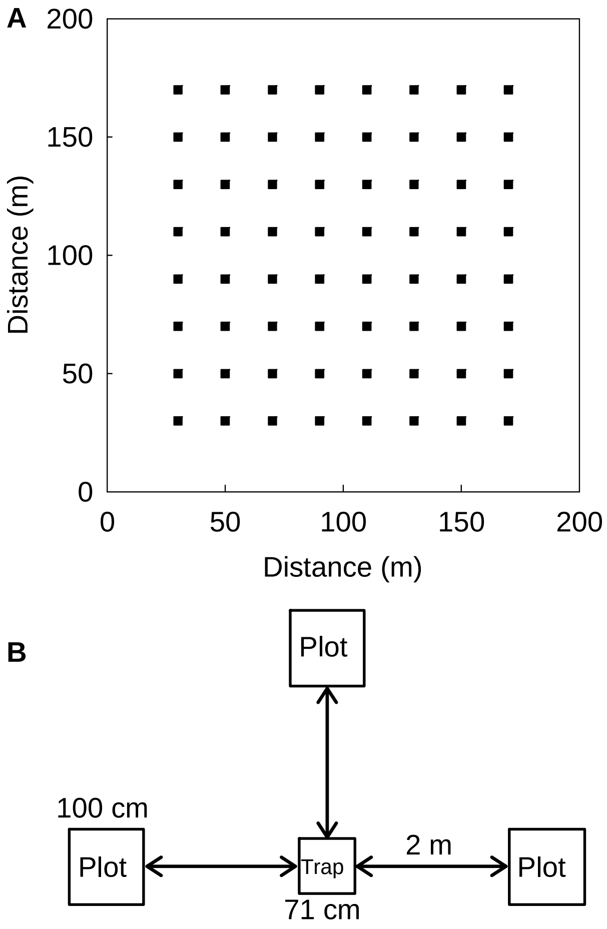

Figure S1. (A) Schematic of census station locations within the 4-ha forest dynamics plot in Hawaiian native forest, and (B) diagram of a census station with three 1-m^2 seedling subplots within 2 m of a 0.5-m^2 seed trap.

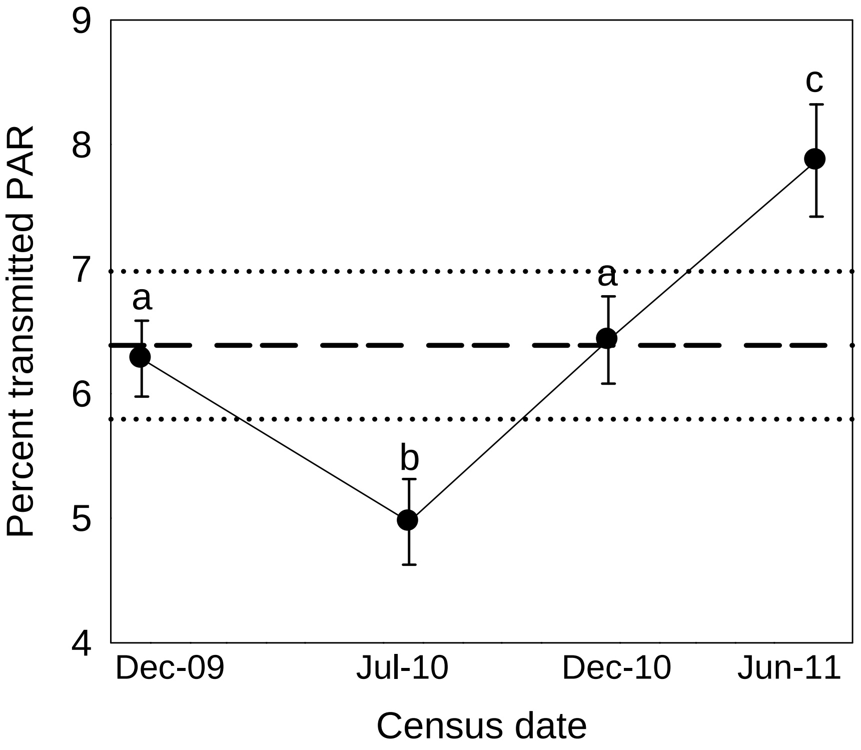

Figure S2. Mean TPAR at each census interval in Hawaiian wet forest, letters based on Tukey's HSD analysis of repeated measures ANOVA; error bars represent SE, TPAR mean (6.4%) and SE over all four censuses represented by dashed horizontal line and represented by dotted horizontal lines, respectively.

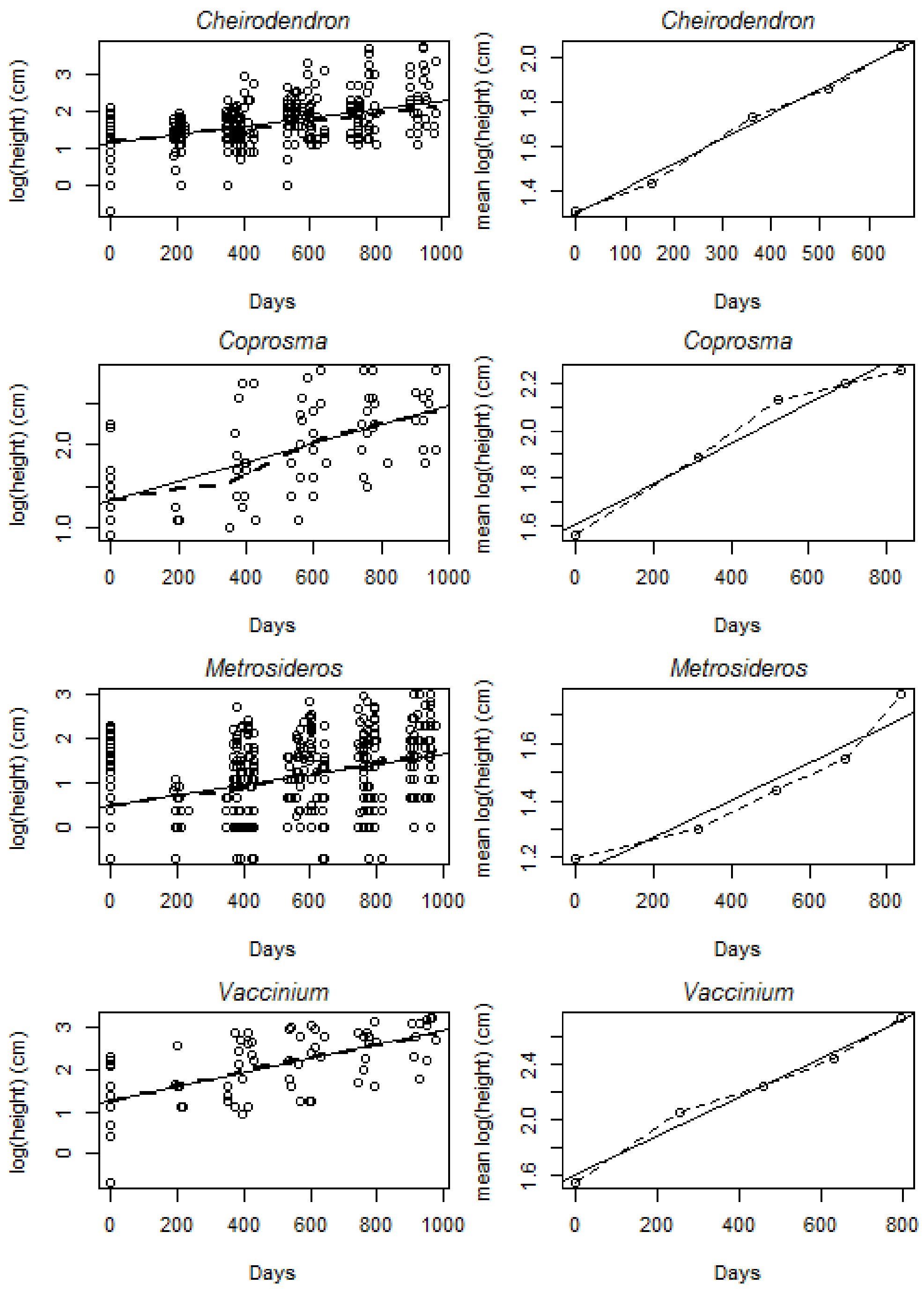

Figure S3. For each species, left plot shows natural log transformed height data over time (days since first measurement) and right plot shows natural log transformed mean heights versus mean days at each census (N = 5 censuses). Solid lines represent log-linear models and dashed lines

represent lowess smoothed lines. Model fits show that log-linear models provide a reasonable approximation of growth for seedling species over the time period examined here.

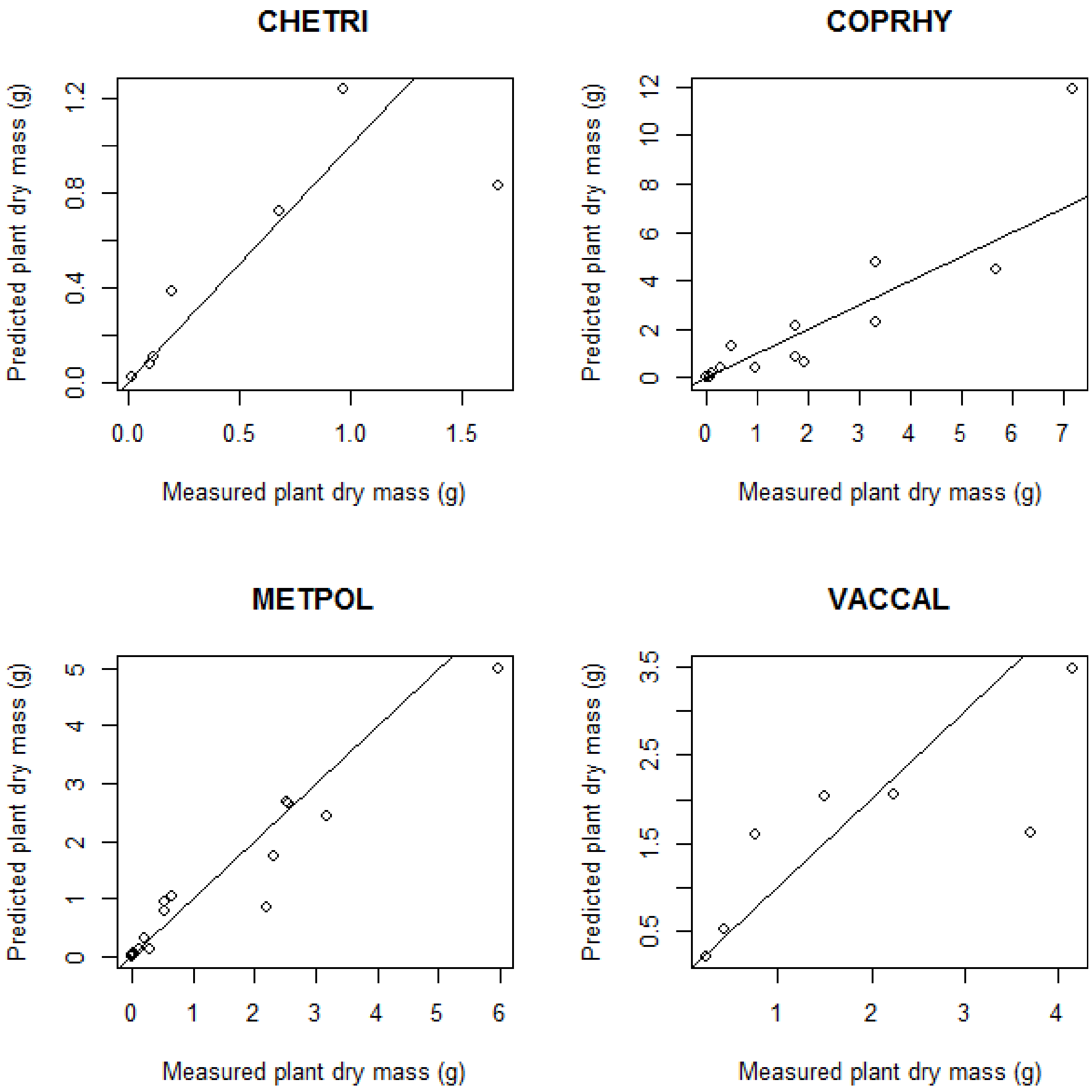

Figure S4A. Scatter plots of actual versus predicted plant mass of seedlings used for allometric equations (see Table S2 for full equations).

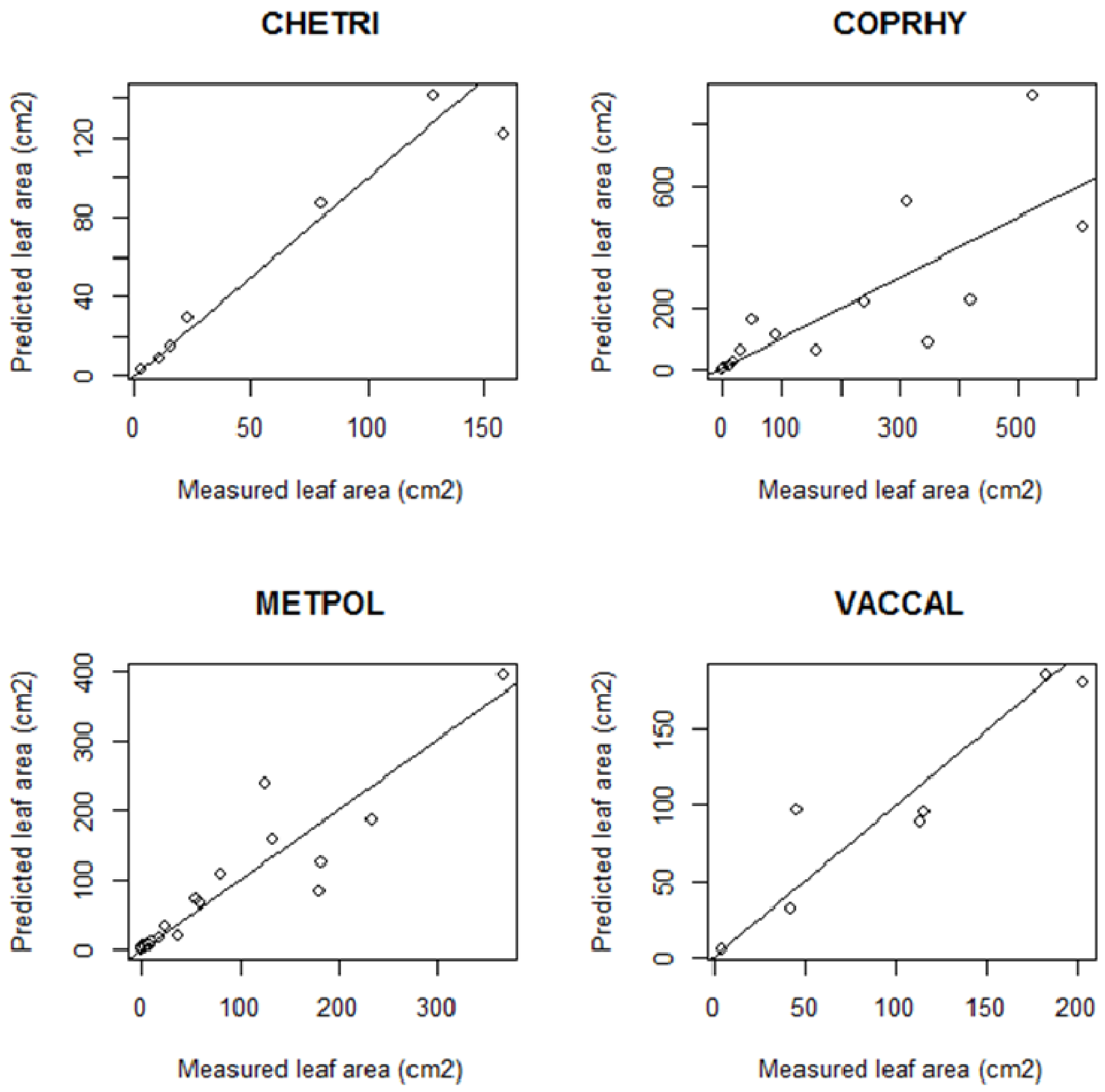

107
108 **Figure S4B.** Scatter plots of actual versus predicted plant mass of seedlings used for allometric
109 equations (see Table S2 for full equations).

110

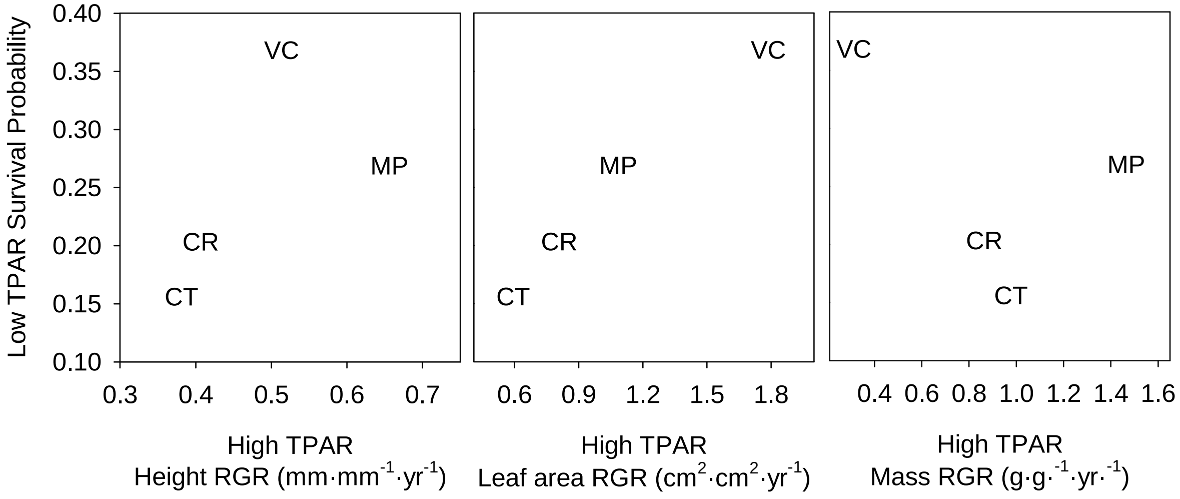

Figure S5. Trade-offs between RGR in high TPAR and survival in low TPAR showing species rank changes in each category (species abbreviations as in Table 1).
